# Macrophage EP4 Deficiency Drives Atherosclerosis Progression via CD36-Mediated Lipid Uptake and M1 Polarization

**DOI:** 10.3390/cells14131021

**Published:** 2025-07-04

**Authors:** Xinyu Tang, Qian Chen, Manli Guo, Ying Wen, Cuiping Jia, Yun Bu, Ting Wang, Yuan Zhang, Waiho Tang

**Affiliations:** 1 School of Medicine, South China University of Technology, Guangzhou 510006, China; 2 Institute of Pediatrics, Guangzhou Women and Children’s Medical Center, Guangzhou Medical University, Guangzhou 510623, China

**Keywords:** EP4, macrophage polarization, foam cell, CD36, atherosclerosis

## Abstract

Atherosclerosis is a chronic inflammatory disease and a major pathological basis of numerous cardiovascular conditions, with a high global mortality rate. Macrophages play a pivotal role in its pathogenesis through phenotypic switching and foam cell formation. Prostaglandin E2 receptor subtype 4 (EP4) highly expressed on the macrophage surface, is involved in various pathophysiological processes, such as inflammation and lipid metabolism. However, the role of macrophage EP4 in the progression of atherosclerosis remains unclear. To determine whether macrophage EP4 affects the progression of atherosclerosis by regulating foam cell formation and macrophage polarization. Myeloid-specific EP4 knockout mice with an ApoE-deficient background were fed a Western diet for 16 weeks. Our results showed that EP4 expression was significantly downregulated during atherosclerosis. EP4 deficiency was found to exacerbate atherosclerotic plaque formation and destabilizes plaques. In vitro studies further demonstrated that loss of EP4 in myeloid cells promoted foam cell formation and M1 macrophage polarization. Both transcriptomic and proteomic analysis showed that EP4 may regulate these processes by regulating CD36 expression in macrophage, which was further confirmed by Western blot and qPCR. In summary, deficiency of EP4 receptor in macrophages enhance foam cell formation and M1 polarization by upregulating CD36 expression, thereby accelerating the progression of atherosclerosis.

## 1. Introduction

Atherosclerosis (AS) is a chronic and progressive vascular disease characterized by the accumulation of plaques within the arterial wall. The plaques, composed of lipids, cholesterol, calcium, cellular debris, and fibrous tissue, gradually narrow and stiffen the arteries, thereby reducing blood flow to vital organs [1]. With a complex pathogenesis, AS serves as the underlying cause of various cardiovascular diseases, including coronary artery disease (e.g., myocardial infarction, cerebrovascular disease (e.g., ischemic stroke), and peripheral artery disease, each contributing substantially to global morbidity, disability, and healthcare burdens [2,3,4]. As a result, AS remains the leading cause of death worldwide, accounting for approximately 17.9 million deaths annually.

Macrophages have been shown to play an important role in the pathogenesis of AS [5]. They engulf oxidized low-density lipoproteins (oxLDL) via scavenger receptors, resulting in intracellular lipid accumulation and foam cell formation hallmark of early atherosclerotic lesions. The process disrupts lipid homeostasis and contributes to AS progression [6,7]. In addition, the inflammatory cytokines secreted by macrophages and the associated immune response also influence the development of plaque. The ratio of M1 and M2 macrophages within the plaques is the key determinant of plaque fate [8,9]. Therefore, elucidating the mechanisms underlying macrophage foam cell formation and polarization is essential for the development of targeted therapies against AS.

Prostaglandin E2 (PGE2) is a biologically active endogenous lipid molecule widely distributed in human tissues, involved in diverse physiological and pathological processes, such as inflammation [10], smooth muscle contraction and relaxation [11], and lipid metabolism [12]. PGE2 exerts its effects through four G protein-coupled receptors termed Prostaglandin E receptor EP1, EP2, EP3 and EP4. Among these, prostaglandin E2 receptor subtype 4 (EP4) is highly expressed in macrophages with high affinity, suggesting that many of the biological effects of PGE2 are mediated through EP4 signaling [13]. EP4 has been found to play a critical role in cardiovascular pathophysiology. EP4 deficiency impairs cardiac function, aggregates cardiomyocyte injury and necrosis [14]; exacerbates inflammatory response, promotes smooth muscle cell apoptosis, and contributes to the development of abdominal aortic aneurysms [15]. In addition, EP4 deficiency has been associated with hypercholesterolemia [16] and impaired cardiac fatty acid metabolism [17], whereas EP4 agonists have been shown to prevent diet-induced hypercholesterolemia [16] and improve cardiomyocyte function [17]. Collectively, these findings suggest that EP4 helps maintain cardiovascular homeostasis by regulating inflammation and lipid metabolism. However, its precise mechanistic role in atherosclerosis needs to be further explored.

In this study, we investigated the role of macrophage EP4 in the development of AS. EP4 expression was found to be downregulated in both atherosclerotic plaques and oxLDL-stimulated macrophages. Macrophage-specific EP4 knockdown promoted atherosclerotic lesion formation. EP4 deficiency enhanced macrophage foam cell formation and M1 polarization at least in part by upregulating CD36 expression.

## 2. Methods

### 2.1. Animals

*EP4^Flox^* mice were generated using the CRISPR-Cas9 system by inserting loxP site flanking exons 2 at Shanghai Model Organisms Center, Inc. (Shanghai, China). Lyz2-Cre mice were purchased from Jackson Laboratory. Myeloid-specific EP4 knockout mice (*EP4^MKO^*) were generated by crossing *EP4^Flox^* mice with Lyz2-Cre mice. *ApoE^−/−^EP4^Flox^* mice were generated by crossing *ApoE^−/^^−^* mice with *EP4^Flox^* mice, and *ApoE^−/^^−^EP4^MKO^* mice were subsequently generated by crossing *ApoE^−/^^−^EP4^Flox^* mice with Lyz2-Cre mice. All genotypes were confirmed by qRT-PCR. All mice were group-housed 3–5/cage in the SPF animal facility. Four-week-old *ApoE^−/^^−^EP4^Flox^* mice and *ApoE^−/^^−^EP4^MKO^* mice were randomly selected for the experiment and assigned to groups, each group consisting of 6–8 mice, then fed either a Western diet (40% kcal% Fat, 1.25% Cholesterol, 0.5% Cholic, D12109C, Research Diets, New Brunswick, NJ, USA) or Chow diet for 16 weeks. For rescue experiments, all mice were fed Western diet for 12 weeks then mice were intraperitoneally injected with Sulfosuccinimidyl oleate sodium (SSO, 25 mg/kg/week, HY-112847A, MCE, Monmouth Junction, NJ, USA), an irreversible inhibitor of CD36 that covalently binds to extracellular lysine residues of the receptor, thereby blocking its ligand-binding capacity, for 4 weeks while continuing the Western diet. The sample size of 6–8 mice per group was determined based on established standards in the field and compliance with the 3Rs principles to ensure both statistical robustness and ethical animal use. The order of removing treatment-group mice from each cage was systematically balanced across all cages to prevent any treatment group from consistently being handled first or last during the extraction process. Investigators were unblinded to treatment groups during compound administration but maintained blinding throughout later experimental stages and statistical evaluation. No survival procedures were performed; euthanasia was conducted under anesthesia without recovery. All animal experiments were approved by the Animal Care and Use Committee of Guangzhou Women and Children’s Medical Center, Guangzhou Medical University, China (RSDW-2023-01397).

### 2.2. Genotyping

Genomic DNA was extracted from 2 mm tail biopsies of 4-week-old mice using the Direct Mouse Genotyping Kit Plus (K1027, APExBio, Houston, TX, USA). PCR was performed to amplify target region, and specific primers were designed to detect mouse alleles. The primer sequences are listed in Table S1.

### 2.3. Macrophage Culture and Treatments

Bone marrow-derived macrophages (BMDMs) were isolated from wild-type (WT), EP4^Flox^, and EP4^MKO^ mice. Briefly, fresh bone marrow cells were cultured in DMEM (C11995500BT, Gibco, Grand Island, NY, USA) with 10% FBS (164210-50, Procell, Wuhan, China), 1% penicillin/streptomycin (15140-122, Gibco, Grand Island, NY, USA), and murine M-CSF (10 ng/mL, 315-02, Peprotech, Cranbury, NJ, USA) for 7 days. Human monocyte-derived macrophages (HMDMs) were isolated from healthy donor PBMCs using magnetic-activated cell sorting (MACS), then differentiated for 7 days in M-CSF-containing medium (20 ng/mL). To ascertain EP4 function, BMDMs were treated with CAY10580 (100 nM, HY-135259, MCE, Monmouth Junction, NJ, USA) for 24 h.

### 2.4. RNA Sequencing (RNA-Seq)

WT BMDMs were either treated with (oxidized low-density lipoprotein) oxLDL or left untreated for 24 h. Total RNA was extracted using Trizol reagent, and RNA libraries were constructed using the NEBNext^®^ Ultra^TM^ RNA Library Prep Kit (E7770, NEB, Ipswich, MA, USA) and NEBNext^®^ Ultra^TM^ Directional RNA Library Prep Kit (E7760s, NEB, Ipswich, MA, USA), according to the manufacturer’s instructions. RNA sequencing was performed on an Illumina platform (NEB, Ipswich, MA, USA). Differential expression analysis was performed using the DESeq2 R package (1.16.1). Genes with an adjusted *p*-value (Padj) < 0.05 were considered differentially expressed. Functional enrichment analysis of the differentially expressed genes in KEGG pathways was performed using the cluster Profiler R package (v4.8.0). Padj < 0.05 was considered significantly enriched.

### 2.5. Proteomic Sequencing

Proteomic analysis was performed on BMDMs isolated from EP4^Flox^ and EP4^MKO^ mice following treatment with oxLDL. Briefly, samples were lysed in SDT buffer (4%SDS, 100 Mm Tris-HCl, Ph 7.6), incubated in boiling water for 15 min, and centrifuged at 14,000× *g* for 15 min. Protein concentrations were determined using the BCA protein assay kit (P0012, Beyotime, Shanghai, China), and samples were stored at −80 °C until further analysis. To assess protein quality, 20 μg of protein from each sample was separated on a 12% SDS-PAGE gel and visualized using Coomassie Blue R-250 staining (BB-3720, Bestbio, Shanghai, China). For proteomic analysis, 100 μg of protein per sample was processed using the filter-aided sample preparation (FASP) method and subsequently lyophilized. The digested peptides were analyzed on a nanoElute UHPLC system (Bruker, Bremen, Germany) coupled to a timsTOF Pro mass spectrometer (Bruker, Bremen, Germany) equipped with a CaptiveSpray source. Raw mass spectrometry data were processed using MaxQuant software (v1.6.17.0, Max Planck Institute of Biochemistry, Martinsried, Germany). MS data were searched against the UniProt database. Label-free quantification (LFQ) was applied to determine based on normalized spectral protein intensities. Proteins with a Fold change >2 or <0.5 and *p* value (Student’s *t* test) < 0.05 were considered deferentially expressed proteins (DEPs). Pathway enrichment analysis was performed using KEGG Orthology And Links Annotation (KOALA) software (v V2.2, Kanehisa Laboratories, Kyoto University, Kyoto, Japan). The KEGG GENES database (version: KO_INFO_END.txt (2022.11.05) was used for aligning target protein sequences, which were subsequently annotated with KO label, and automatically classified into associated pathways.

### 2.6. Foam Cell Induction and Uptake Assay

For foam cell induction, BMDMs and HMDMs were incubated with oxLDL (50 μg/mL, YB-002, Yiyuan biotechnologies, Guangzhou, China) for 24 h. Cells were then fixed and stained with Oil Red O (G1015, Servicebio, Wuhan, China), and lipid accumulation was observed by light microscopy. For the ox-LDL uptake assay, cells were serum-starved in DMEM for 12 h, followed by incubation with Dil-labeled oxLDL (50 μg/mL, YB-001, Yiyuan biotechnologies, Guangzhou, China) for 4 h. Cells were then fixed with 4% paraformaldehyde and stained with hoechst (H3570, Thermo Scientific, Waltham, MA, USA) for nuclei visualization. Fluorescence images were observed using confocal microscopy.

### 2.7. Macrophage Polarization

To induce macrophage polarization, cells were cultured in complete DMEM (10% FBS). BMDMs were stimulated for 24 h under the following conditions: for M1 polarization, lipopolysaccharide (LPS, 100 ng/mL, Sigma, Burlington, MA, USA) and IFN-γ (20 ng/mL, 315-05, Peprotech, Cranbury, NJ, USA); for M2 polarization, IL4 (20 ng/mL, 214-14, Peprotech, Cranbury, NJ, USA) and IL13 (20 ng/mL, 210-13, Peprotech, Cranbury, NJ, USA). Expression of the phenotypic markers was analyzed by qRCR for TNF-α, Arg1, etc., and by flow cytometry using surface markers CD86 (M1) and CD206 (M2).

### 2.8. Histological Analyses

For en face staining of atherosclerotic plaques, aortas were isolated from ApoE^−/−^EP4^Flox^ mice and ApoE^−/−^EP4^MKO^ mice after 16 weeks of Western diet feeding or Chow diet feeding. Then aortas were fixed in 4% paraformaldehyde for 24 h and stained with Oil Red O. En face images were acquired by light microscopy and analyzed using Image J software (v 1.53, NIH, Bethesda, MD, USA). For cross-sectional analysis, mouse aortic roots were fixed in 4% paraformaldehyde (G1101, Servicebio, Wuhan, China), embedded in Tissue-Tek O.C.T. compound (4583, SAKURA, Tokyo, Japan), and sliced into 6 μm sections. The sections were stained with hematoxylin and eosin (H&E), Masson’s trichrome, and Oil Red O. Histological images were acquired using a light microscope (Leica DM4, Wetzlar, Germany) and analyzed by Image J software (v 1.53, NIH, Bethesda, MD, USA).

### 2.9. Immunofluorescence

For immunofluorescence staining of cultured macrophages, BMDMs and HMDMs were seeded into glass-bottomed culture dishes at a density of 2 × 10^5^ per well and incubated with oxLDL for 24 h. Cells were then fixed with 4% paraformaldehyde and incubated overnight at 4 °C with the following primary antibodies: Anti-CD68 (1:100, 25747-1-AP, Proteintech, Wuhan, China) and Anti-EP4 (1:100, 24895-1-AP, Proteintech, Wuhan, China). The next day, cells were incubated with Alexa Fluor 594-conjugated IgG antibody (1:200, ab150116, Abcam, Cambridge, UK) and Fluor 488-conjugated IgG antibody (1:200, ab150077, Abcam, Cambridge, UK) for 1 h at room temperature. Nuclei were stained with Hoechst (1:2000, H3570, Thermo Scientific, Waltham, MA, USA) and the images were captured using a Leica SP8 confocal microscope (Leica, Wetzlar, Germany), and analyzed with Image J software (v 1.53, NIH, Bethesda, MD, USA).

For immunostaining of aortic tissue sections, frozen sections were blocked with PBS (SH30256.01B, Cytiva, Marlborough, MA, USA) containing 5% normal goat serum (16210-64, Gibco, Grand Island, NY, USA) and 5% bovine serum albumin (B2064-100G, Sigma, Burlington, MA, USA) for 1 h at room temperature. Sections were then incubated overnight at 4 °C with the following primary antibodies: anti-EP4, anti-CD36 (18836-1-AP, Proteintech, Wuhan, China), anti-Arg1 (16001-1-AP, Proteintech, Wuhan, China), anti-F4/80 (GB113373-100, Servicebio, Wuhan, China), anti-α-SMA (ab7818, Abcam, Cambridge, UK). The next day, the slides were incubated with Alexa Fluor 488-conjugated IgG secondary antibody (1:200, ab150077, Abcam, Cambridge, UK) or Alexa Fluor 594-conjugated IgG secondary antibody (1:200, ab150160, Abcam, Cambridge, UK) at 37 °C for 2 h. Nuclei were visualized with DAPI (H-1200, Vector Laboratories, Burlingame, CA, USA). Immunofluorescence images were captured by Leica SP8 confocal microscopy (Leica, Wetzlar, Germany). Fluorescence intensity quantification was performed using ImageJ software (v 1.53, NIH, Bethesda, MD, USA). The mean fluorescence intensity (MFI) for each region of interest (ROI) was calculated, with group-level MFI representing the average of all matched sections.

### 2.10. Flow Cytometry Analysis

BMDMs were resuspended in PBS with 2% FBS. To assess macrophage polarization following stimulation, cells were first incubated with anti-Mouse CD16/CD32 monoclonal antibody (1:200, 14-0161-85, Invitrogen, Carlsbad, CA, USA) for 15 min to block Fc receptors, then were stained with anti-CD11b-PE-Cy7 (1:200, 101216, Biolegend, San Diego, CA, USA), anti-F4/80-FITC (1:200, 123108, Biolegend, San Diego, CA, USA), anti-CD86-PE (1:200, 105007, Biolegend, San Diego, CA, USA), and anti-CD206-APC (1:200, 321110, Biolegend, San Diego, CA, USA) for 30 min on ice. Then, cell suspensions were filtered through a cell strainer-cap FACS tube and vortex-mixed at medium speed for 10 s immediately before flow cytometry analysis to ensure uniform cell distribution. Data were analyzed by Flowjo software (v 10.6.2, BD, Franklin Lakes, NJ, USA).

### 2.11. RNA Extraction and RT-qPCR

Total RNA was extracted from cells using a RNeasy kit (74104, Qiagen, Hilden, Germany), according to the manufacturer’s instructions. Then, the extracted RNAs were reverse-transcribed using a PrimeScriptTM RT kit (RR037A, Takara, Kusatsu City, Japan). Quantitative real-time PCR was performed using the SYBR-Green (RR820A, Takara, Kusatsu City, Japan) on Applied Biosystems Q6 FastReal-Time PCR System sequencer detector (Thermo Scientific, Waltham, MA, USA). The primers are listed in Table S2. Gene expression was normalized to mouse β-actin or human GAPDH.

### 2.12. Western Blot Analysis

Total protein was extracted using radioimmunoprecipitation assay lysis buffer (P0013, Beyotime, Shanghai, China) supplemented with a protease inhibitor cocktail (539131, Merck, Darmstadt, Germany). Protein concentrations were determined using a BCA Assay Kit (P0009, Beyotime, Shanghai, China). Equal amounts of protein lysates were separated on 10% SDS-PAGE gels and transferred to PVDF membranes. Following blocking with 5% skimmed milk in TBST for 1 h at room temperature, the membranes were incubated overnight at 4 °C with the following primary antibodies: anti-EP4 (1:1000, 24895-1-AP, Proteintech, Wuhan, China), anti-CD36 (1:2000, ab133625, Abcam, Cambridge, UK), anti-Arg1 (1:1000, 16001-1-AP, Proteintech, Wuhan, China), anti-iNOS (1:1000, ab178945, Abcam, Cambridge, UK), anti-Hsp90 (1:10,000, ab203126, Abcam, Cambridge, UK), anti-GAPDH (1:10,000, ab8245, Abcam, Cambridge, UK). After washing, membranes were incubated with the secondary antibodies for 1 h at room temperature. Protein bands were visualized using an enhanced chemiluminescence (ECL) detection kit (WBKLS0500, Merck, Darmstadt, Germany) and quantified using ImageLab software (v 4.0, Bio-rad, Hercules, CA, USA).

### 2.13. siRNA Transfection

Mouse CD36 siRNA and human EP4 siRNA were transiently transfected into cells using Lipofectamine^TM^ RNAiMAX Transfection Reagent (13778150, Thermo Fisher, Waltham, MA, USA), according to the manufacturer’s instructions. A non-targeting siRNA was used as a negative control (Table S3). The knockdown efficiency was assessed by quantitative PCR and Western blot analysis.

### 2.14. Statistical Analysis

Data were presented as mean ± SEM, and the statistical analysis was performed using GraphPad Prism software (v8.3.0, GraphPad Software, San Diego, CA, USA). Exclusion criteria included spontaneous death or failure to meet experimental endpoints. The normality of data was checked using the Shapiro–Wilk normality test. For comparison with two groups, an unpaired two-tailed Student’s *t*-test was used if the data passed the normality test. For comparisons involving more than two groups, One-way ANOVA followed by Tukey’s multiple comparisons was used. Two-way ANOVA with Tukey’s multiple comparisons test was employed for multifactorial comparisons involving three or more groups. The difference with *p* < 0.05 was considered statistically significant.

## 3. Results

### 3.1. Macrophage EP4 Expression Was Reduced During Atherosclerosis

**Figure 1:**
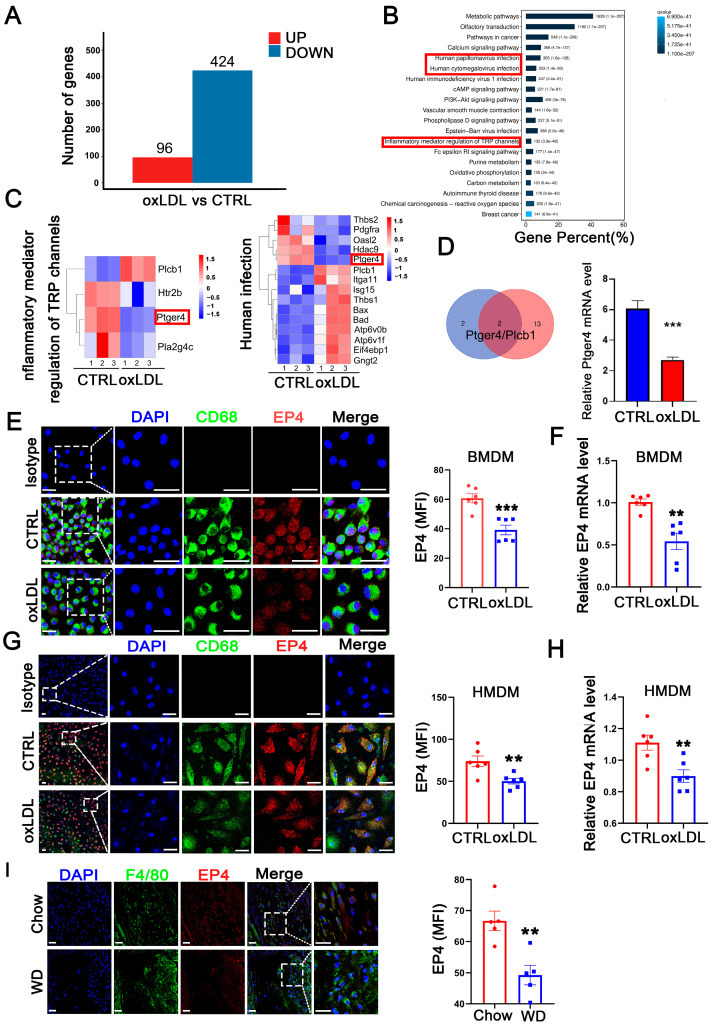
Macrophage EP4 expression was reduced in oxLDL-treated macrophages and atherosclerotic lesions. BMDMs were stimulated with oxLDL for 24 h and collected for RNA sequencing. (**A**) The histogram depicting DEGs in wild-type BMDMs treated with or without oxLDL. (**B**) KEGG pathway enrichment analysis of DEGs. (**C**) The gene sets enriched in each KEGG inflammation-related pathway. (**D**) Schematic diagram illustrating the overlap of DEGs among KEGG inflammation-related signaling pathways. Bar graph showing downregulation of *ptger4 (EP4*) expression following treatment with oxLDL. Immunofluorescence staining of EP4 (red) and CD68 (green) in BMDMs (**E**) and HMDMs (**G**) cultured with oxLDL (50 μg/mL) for 24 h was performed. Nuclei were stained with DAPI (blue). The fluorescence intensity of EP4 was quantified by ImageJ (*n* = 6). Scale bar: 20 μm. Quantitative RT-PCR analysis of *EP4* mRNA expression in BMDMs (**F**) and HMDMs (**H**) cultured with oxLDL (50 μg/mL) for 24 h (*n* = 6). (**I**) Immunofluorescence staining of EP4 (red) and CD68 (green) within the plaque lesions from ApoE^−/−^ mice after 16 weeks of Western diet or Chow diet (*n* = 5). EP4 fluorescence intensity was quantified by ImageJ. Nuclei were stained with DAPI (blue). Scale bar: 20 μm. Data are presented as mean ± SEM, ** *p* < 0.01, *** *p* < 0.001 versus control group, Unpaired *t*-test. DEGs: differentially expressed genes; BMDM: bone marrow-derived macrophage; HMDM: human monocyte-derived macrophage; oxLDL: oxidized low-density lipoprotein; WD: Western diet.

To investigate genes potentially involved in the pathological progression of atherosclerosis, we established an in vitro cell model using bone marrow-derived macrophages (BMDM) stimulated with oxidized low-density lipoprotein (oxLDL). Following 24 h of oxLDL stimulation, BMDMs were collected for RNA-sequencing. Compared to the control group, a total of 520 differentially expressed genes (DEGs) were identified, including 96 upregulated genes and 424 downregulated genes (Figure 1A). Subsequent gene set enrichment analysis (GSEA) and KEGG pathway enrichment analysis revealed that these DEGs were significantly enriched in inflammatory response pathways, such as human cytomegalovirus infection, human papillomavirus infection, and inflammatory mediator regulation of TRP channels (Figure 1B). By intersecting the gene sets enriched in the three pathways, two candidate genes, *Ptger4* (*EP4*) and *plcb1*, were identified (Figure 1C,D), among which *EP4* exhibited the highest fold change in RNA sequencing. The downregulation of the EP4 protein (Figure 1E,G) and mRNA (Figure 1F,H) was further confirmed in both BMDMs and HMDMs following oxLDL stimulation in vitro. To confirm the findings in vivo, atherosclerosis was induced in *ApoE^−/−^* mice by feeding a Western diet for 16 weeks. Immunofluorescence staining revealed significantly reduced EP4 expression in macrophages from Western diet-fed mice, compared to those fed on Chow diet (Figure 1I). Collectively, these results indicate that EP4 expression is downregulated in macrophages during atherogenesis.

### 3.2. Macrophage EP4 Deficiency Exacerbated Atherosclerosis and Destabilizes Plaques

**Figure 2:**
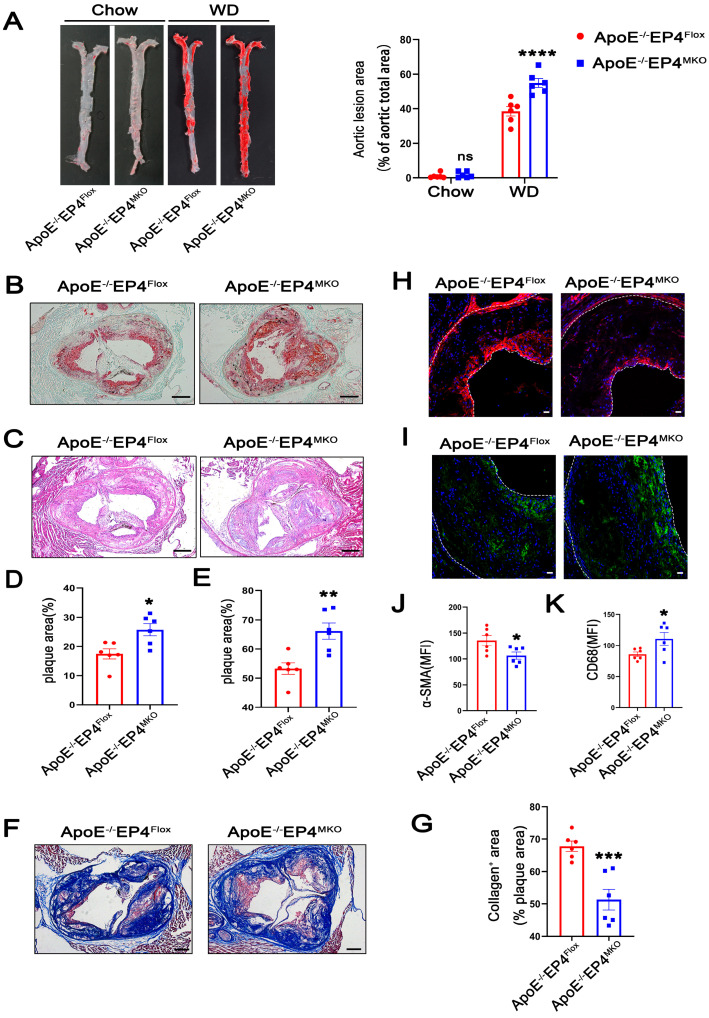
Macrophage EP4 deficiency promoted plaque formation and instability in atherosclerosis. *ApoE^−/−^EP4^Flox^* and *ApoE^−/−^EP4^MKO^* mice were fed either with Western diet or Chow diet for 16 weeks. Total aorta and aortic roots were collected. (**A**) Representative En face images and quantification of Oil Red O-stained aorta from *ApoE^−/−^EP4^Flox^* and *ApoE^−/−^EP4^MKO^* mice (Chow: *n* = 6; WD: *n* = 6). Representative images and quantification of Oil Red O staining ((**B**,**D**), scale bar: 200 μm), H&E staining ((**C**,**E**), scale bar: 200 μm), Masson’s trichrome staining ((**F**,**G**), scale bar: 200 μm) in aortic root sections from *ApoE^−/−^EP4^Flox^* and *ApoE^−/−^EP4^MKO^* mice after 16 weeks of Western diet. Immunofluorescence staining and quantitation of α-SMA (the smooth muscle cell marker) (**H**,**J**) and CD68 (the macrophage marker) (**I**,**K**) in the aortic roots from *ApoE^−/−^EP4^Flox^* and *ApoE^−/−^EP4^MKO^* mice, the fluorescence intensity was quantified by ImageJ (*n* = 6), α-SMA stained as red, CD68 as green, nuclei as blue. Scale bar: 20 μm. Data presented as mean ± SEM, ns: not significant, * *p* < 0.05, ** *p* < 0.01, *** *p* < 0.001, **** *p* < 0.0001, versus the ApoE^−/−^EP4^Flox^ mice, two-way ANOVA in (**A**), Unpaired *t*-test in (**D**,**E**,**G**,**J**,**K**). WD: Western diet.

To further investigate the role of macrophage EP4 in atherosclerosis, we generated myeloid-specific EP4 knockout mice on an *ApoE^−/^^−^* background (*ApoE^−/^^−^ EP4^MKO^* mice) (Figure S1A–C). Efficient Cre-mediated recombination at the locus of *Ptger4* was confirmed in BMDMs isolated from *EP4^MKO^* mice (Figure S1D,E). Notably, compared to *ApoE^−/^^−^EP4^Flox^* mice, *ApoE^−/^^−^ EP4^MKO^* mice exhibited significantly larger atherosclerotic plaque areas (Figure 2A,C,E) and increased lipid deposition (Figure 2B,D) within the aortic root, suggesting that EP4 deficiency exacerbates plaque formation and intraplaque lipid accumulation.

Plaque stability is a critical determinant of clinical outcomes during atherosclerosis, as unstable plaques are prone to rupture, leading to thrombosis and acute cardiovascular events [18]. To investigate whether macrophage EP4 deficiency affects plaque stability, collagen content within plaques was examined using Masson’s trichrome staining (red, muscle fibers; blue, collagen). As shown in Figure 2F,G, ApoE^−/−^EP4^MKO^ mice showed a significant reduction in collagen content, suggesting compromised plaque stability. In addition, plaques from *ApoE^−/^^−^EP4^MKO^* mice displayed decreased expression of α-SMA (Figure 2H,J) and increased infiltration of CD68-positive macrophages (Figure 2I,K). Collectively, these data demonstrate that macrophage EP4 deficiency promotes atherosclerotic plaque progression and destabilization, highlighting its critical role in both lesion development and plaque stability.

### 3.3. EP4 Deficiency Promoted Macrophage M1 Polarization Both In Vitro and In Vivo

**Figure 3:**
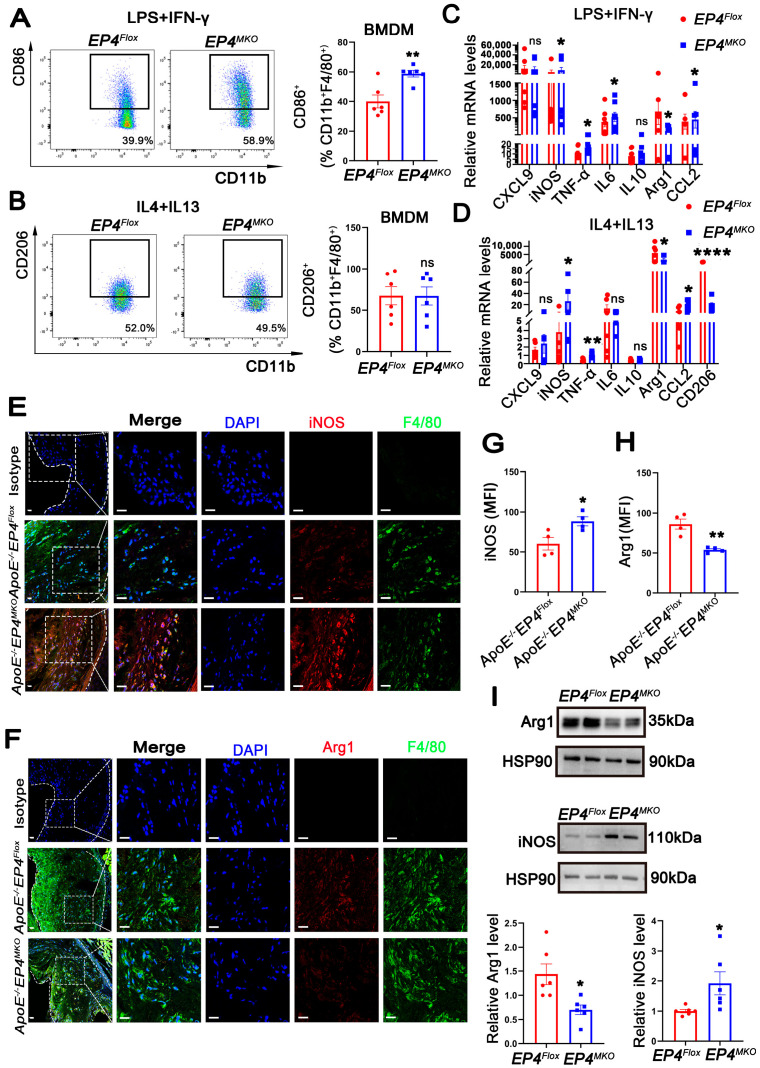
EP4 deficiency promoted macrophage M1 polarization both in vitro and in vivo. (**A**,**B**) Flow cytometry analysis of CD86 and CD206 expression in BMDMs from *EP4^Flox^* and *EP4^MKO^* mice following stimulation with LPS/IFNγ or IL4/IL13 (*n* = 6). (**C**,**D**) The mRNA level of inflammation cytokines and macrophage polarization markers in BMDMs from *EP4^Flox^* and *EP4^MKO^* mice after stimulation with LPS/IFNγ or IL4/IL13 was determined by qRT-PCR (*n* = 6). Immunofluorescence staining and quantification of iNOS (**E**,**G**) and Arg1 (**F**,**H**) in the aortic roots from *ApoE^−/−^EP4^Flox^* and *ApoE^−/−^EP4^MKO^* mice after 16 weeks of Western diet; the fluorescence intensity was quantified by ImageJ (*n* = 4). Nuclei were stained with DAPI (blue); iNOS or Arg1 was stained red; F4/80 as green. Scale bar: 20 μm. (**I**) Western blot analysis and quantification of Arg1 and iNOS expression in BMDMs from *EP4^Flox^* and *EP4^MKO^* mice (*n* = 6). Data presented as mean ± SEM, ns: not significant, * *p* < 0.05, ** *p* < 0.01, **** *p* < 0.0001 versus the *ApoE^−/−^EP4^Flox^* mice or *EP4^Flox^* mice, Unpaired *t*-test. BMDM: bone marrow-derived macrophage.

Macrophage phenotypic switching plays a critical role in the progression of atherosclerosis. Accumulating evidence indicates that unstable plaques are enriched with pro-inflammatory M1 macrophages, whereas stable plaques predominantly contain anti-inflammatory M2 macrophages [19]. To determine whether macrophage EP4 deficiency affects phenotypic switching, *EP4^MKO^* BMDMs were subjected to M1- (LPS/IFN-γ) or M2- (IL4/IL13) polarizing conditions. Upon LPS/IFN-γ stimulation, *EP4^MKO^* BMDMs exhibited increased CD86 expression, a surface marker of M1 macrophages (Figure 3A), whereas CD206 (M2 surface marker) remained unchanged upon IL4/IL13 stimulation (Figure 3B). RT-qPCR analysis further confirmed upregulation of M1-associated genes (*iNOS*, *TNFα*, *IL6*, and *CCL2*) in *EP4^MKO^* BMDMs upon LPS/IFN-γ stimulation (Figure 3C), and downregulation of M2 markers (*CD206*, *Arg-1*) upon IL4/IL13 stimulation (Figure 3D). Western blot analysis corroborated these findings, showing increased iNOS and decreased Arg-1 protein levels in *EP4^MKO^* BMDMs (Figure 3I).

In vivo, aortic root sections from *ApoE^−/^^−^EP4^MKO^* mice and *ApoE^−/−^EP4^Flox^* mice fed a Western diet for 16 weeks were analyzed. Immunofluorescence staining revealed increased expression of iNOS (M1 marker) (Figure 3E,G) and reduced Arg1 (M2 marker) (Figure 3F,H) in plaques from *ApoE^−/^^−^EP4^MKO^* mice, indicating a shift toward M1 polarization. Taken together, these data demonstrate that macrophage-specific EP4 deficiency promotes M1-polarization both in vitro and in vivo, contributing to a pro-inflammatory phenotype in atherosclerotic lesions.

### 3.4. Macrophage EP4 Deficiency Enhanced Foam Cell Formation

**Figure 4:**
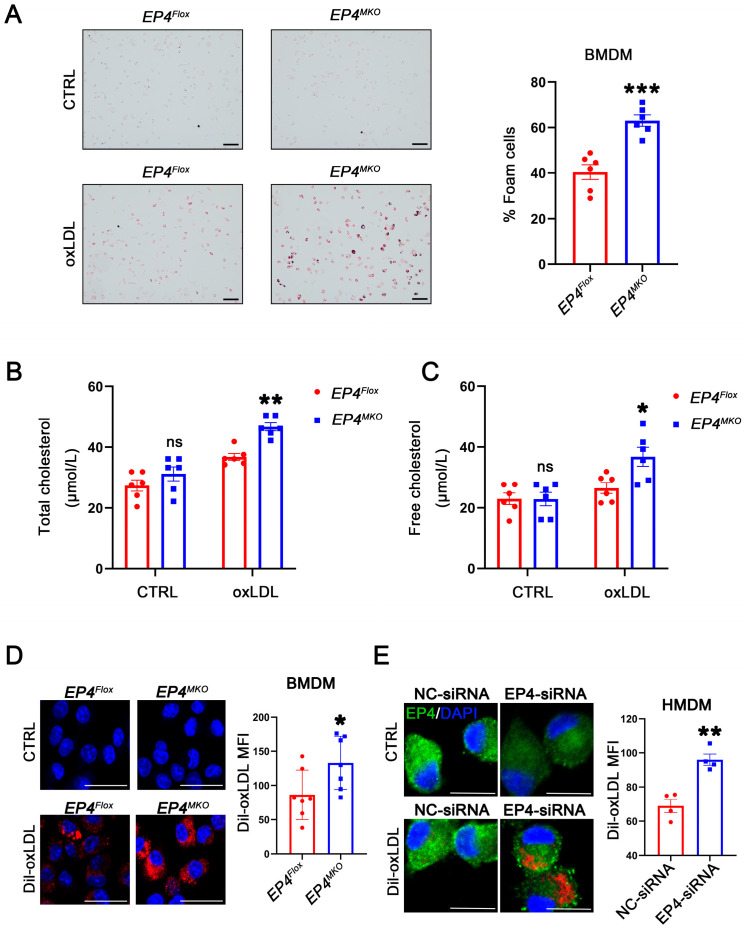
Macrophage EP4 deficiency promotes foam cell formation. (**A**) Representative images and quantitation of Oil Red O-stained foam cells in *EP4^Flox^* and *EP4^MKO^* BMDMs after culture with or without oxLDL (50 μg/mL) (*n* = 6), scale bar: 50 μm. (**B**,**C**) Quantification of total cholesterol and free cholesterol accumulation in *EP4^Flox^* and *EP4^MKO^* BMDMs treated with or without oxLDL for 24 h (*n* = 6). Representative images and quantitation of Dil-labeled oxLDL uptake in BMDMs (**D**) (*n* = 7) and HMDMs (**E**) (*n* = 4). Nuclei were stained with DAPI (blue); EP4 was stained green. The fluorescence intensity was quantified by ImageJ, scale bar: 20 μm. Data presented as mean ± SEM, ns: not significant, * p < 0.05, ** *p* < 0.01, *** p < 0.001, versus EP4^Flox^ BMDMs or NC-siRNA transfected HMDMs, Unpaired *t*-test. BMDM: bone marrow-derived macrophage; HMDM: human monocyte-derived macrophage.

To investigate the potential role of EP4 in foam cell formation, BMDMs derived from *EP4^MKO^* and *EP4^Flox^* mice were treated with oxLDL. Oil Red O staining showed that EP4 deficiency significantly enhanced foam cell formation and intracellular lipid accumulation compared to *EP4^Flox^* BMDMs (Figure 4A–C). To determine whether this phenotype was driven by increased uptake of modified lipoproteins, a Dil-labeled oxLDL uptake assay was performed. Immunofluorescence analysis showed that increased internalization of Dil-oxLDL in *EP4^MKO^* BMDMs compared to *EP4^Flox^* controls (Figure 4D). To validate these findings in human cells, HMDMs were transfected with EP4-siRNA (Figure S2). EP4 knockdown significantly increased uptake of Dil-oxLDL in HMDMs compared to those transfected with negative control (NC)-siRNA (Figure 4E). Collectively, these data suggest that EP4 deficiency enhances ox-LDL uptake and promotes foam cell formation in both murine and human macrophages.

### 3.5. Activation of EP4 Suppresses Macrophage M1 Polarization and Foam Cell Formation

**Figure 5:**
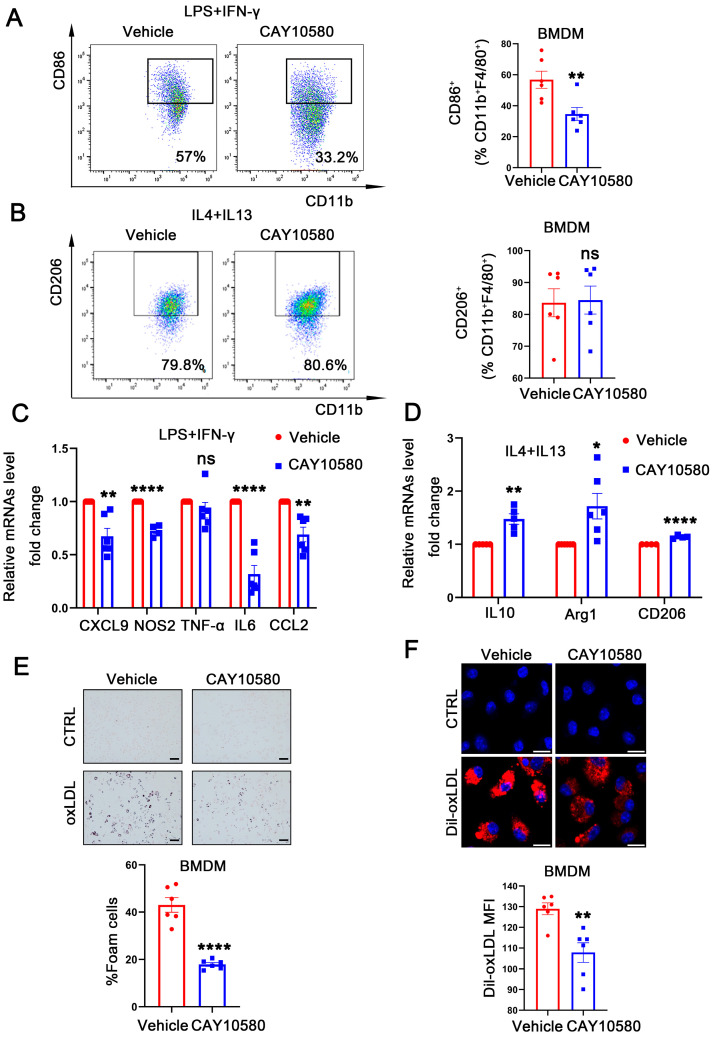
Activation of EP4 suppressed macrophage M1 polarization and foam cell formation. (**A**,**B**) Flow cytometry analysis of CD86 and CD206 expression in BMDMs treated with Vehicle (0.00068% ethanol) or CAY10580 (100 nM) (*n* = 6). (**C**,**D**) qRT-PCR was used to determine the mRNA levels of pro-inflammatory cytokines and macrophage polarization markers in BMDMs pretreated with Vehicle (0.00068% ethanol) or CAY10580 (100 nM), followed by stimulation with LPS/IFN-γ or IL4/IL13 (*n* = 6). (**E**) Oil Red O staining and quantification of lipid accumulation in WT BMDMs pre-treated with Vehicle (0.00068% ethanol) or CAY10580 (100 nM) followed by stimulation with or without oxLDL (50 μg/mL) for 24 h (*n* = 6), scale bar: 50 μm; (**F**) representative images and quantitation of Dil-labeled oxLDL uptake in BMDMs pretreated with Vehicle (0.00068% ethanol) or CAY10580 (*n* = 6), scale bar: 20 μm; data presented as mean ± SEM, ns: not significant, * *p* < 0.05, ** *p* < 0.01, **** *p* < 0.0001, Unpaired *t*-test, BMDM: bone marrow-derived macrophage.

To further explore the functional role of macrophage EP4 in atherosclerosis, WT BMDMs were pre-treated with CAY10580, followed by stimulation with LPS + IFN-γ and IL4 + IL13. Flow cytometry analysis showed a decreased proportion of CD86^+^ macrophages after CAY10580 treatment (Figure 5A), whereas the proportion of CD206^+^ macrophages remained unchanged (Figure 5B). Consistent with these findings, qRT-PCR analysis showed that CAY10580 treatment attenuated expression of M1-associated pro-inflammatory factors upon LPS and IFN-γ stimulation (Figure 5C) while enhancing the expression of M2-associated anti-inflammatory factors following IL4 and IL13 stimulation (Figure 5D).

Then, WT BMDMs were pre-treated with CAY10580, followed by stimulation with oxLDL. Compared to cells stimulated with oxLDL alone, pre-activation of EP4 with CAY10580 significantly reduced foam cell formation (Figure 5E) and decreased Dil-oxLDL uptake (Figure 5F). These results indicate that pharmacological activation of EP4 suppresses foam cell formation and M1 polarization, thereby promoting an anti-inflammatory macrophage phenotype, which may potentially ameliorate atherosclerosis.

### 3.6. EP4 Upregulates the Expression of CD36

**Figure 6:**
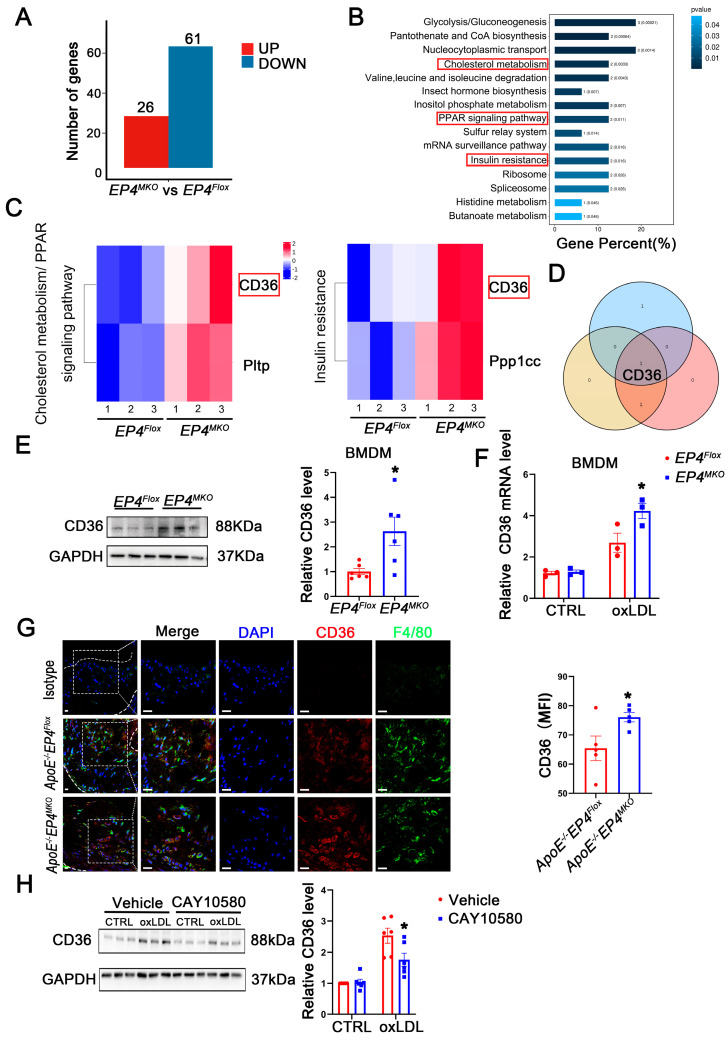
EP4 upregulated the expression of CD36. (**A**) The histogram depicts the number of DEGs between BMDMs from *EP4^Flox^* and *EP4^MKO^* mice after treatment with oxLDL. (**B**) The KEGG pathway enrichment analysis of DEGs. (**C**) The gene sets enriched in KEGG pathways related to inflammation and lipid metabolism. (**D**) Schematic diagram illustrating the overlap of DEGs across KEGG inflammation and lipid metabolism-related signaling pathways. (**E**) Western blot analysis and quantification of CD36 expression in BMDMs from *EP4^Flox^* and *EP4^MKO^* mice after treatment with oxLDL (50 μg/mL) for 24 h (*n* = 6). (**F**) qRT-PCR analysis of CD36 mRNA expression in oxLDL-stimulated BMDMs from the *EP4^Flox^* and *EP4^MKO^* mice (*n* = 3). (**G**) Double-immunofluorescence staining for F4/80 and CD36 in the aortic roots from *ApoE^−/−^EP4^Flox^* or *ApoE^−/−^EP4^MKO^* mice after 16 weeks of Western diet (*n* = 5). Nuclei were stained with DAPI (blue); F4/80 stained as green; CD36 as red, scale bar: 20 μm. (**H**) Western blot analysis and quantification of CD36 expression in BMDMs from WT mice pre-treated with or without CAY10580 (100 nM), followed by stimulation with or without oxLDL (50 μg/mL) for 24 h (*n* = 6). Data presented as mean ± SEM, ns: not significant, * *p* < 0.05, Unpaired *t*-test in (**E**,**G**), two-way ANOVA in (**G**,**H**). DEGs: differentially expressed genes; BMDM: bone marrow-derived macrophage; oxLDL: oxidized low-density lipoprotein.

To elucidate the mechanism by which macrophage EP4 deficiency promotes M1 polarization and foam cell formation, RNA-sequencing (Figure S3) and proteomic profiling were performed on *EP4^MKO^* and *EP4^Flox^* BMDMs following 24 h of oxLDL stimulation. Compared to *EP4^Flox^* BMDMs, *EP4^MKO^* BMDMs exhibited significant alterations in 87 proteins, including 26 upregulated and 61 downregulated (Figure 6A). Subsequent KEGG pathway enrichment analysis identified three pathways associated with inflammatory response and lipid metabolism (Figure 6B,C). Intersection analysis of the enriched proteins revealed CD36 as the sole candidate protein (Figure 6D). The upregulation of CD36 in *EP4^MKO^* BMDMs upon oxLDL stimulation was further validated by Western blotting (Figure 6E) and qRT-PCR (Figure 6F). Consistently, immunofluorescence staining confirmed elevated CD36 expression in aortic plaques from *ApoE^−/^^−^EP4^MKO^* mice compared to *ApoE^−/^^−^EP4^Flox^* mice (Figure 6G), suggesting EP4 deficiency exacerbates atherosclerosis through CD36-mediated inflammatory and metabolic dysregulation.

To further explore the regulatory relationship between CD36 and EP4, BMDMs were pretreated with CAY10580, followed by stimulation with oxLDL. Western blotting analysis revealed that oxLDL significantly increased CD36 expression; such alteration was attenuated by treatment with CAY10580 (Figure 6H), demonstrating EP4-dependent regulation of CD36.

### 3.7. EP4 Deficiency Enhanced Macrophage Foam Cell Formation and M1 Polarization at Least in Part by Upregulating CD36 Expression

**Figure 7:**
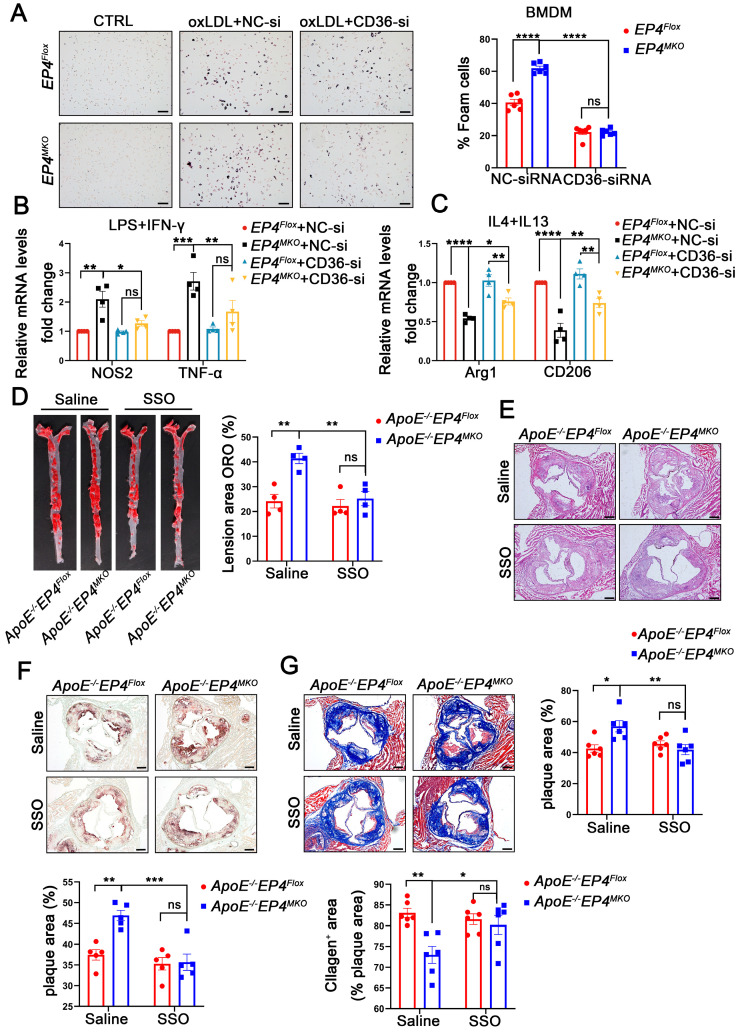
EP4 deficiency enhanced macrophage foam cell formation and M1 polarization at least in part by upregulating CD36 expression. *EP4^Flox^* and *EP4^MKO^* BMDMs were pre-transfected with siRNA-CD36. (**A**) Oil Red O staining and quantification of foam cells in BMDMs after stimulation with or without oxLDL (50 μg/mL) for 24 h (*n* = 6), scale bar: 50 µm. (**B**,**C**) qRT-PCR was used to determine the mRNA levels of pro-inflammatory cytokines and macrophage polarization markers in BMDMs after stimulation with LPS/IFNγ or IL4/IL13 (*n* = 4). *ApoE^−/−^EP4^Flox^* and *ApoE^−/−^EP4^MKO^* mice were fed a Western diet for 12 weeks, followed by intraperitoneally injected with SSO for 4 weeks. The total aorta and aortic roots were collected. (**D**) Representative En face images and quantitation of Oil Red O-stained aorta from *ApoE^−/−^EP4^Flox^* and *ApoE^−/−^EP4^MKO^* mice (*n* = 4). Representative images and quantification of H&E staining ((**E**), *n* = 6, scale bar: 200 μm), Oil Red O staining ((**F**), *n* = 5, scale bar: 200 μm), Masson’s trichrome staining ((**G**), *n* = 6, scale bar: 200 μm) in aortic root sections from *ApoE^−/−^EP4^Flox^* and *ApoE^−/−^EP4^MKO^* mice. Data presented as mean ± SEM, ns: not significant, * *p* < 0.05, ** *p* < 0.01, *** *p* < 0.001, **** *p* < 0.0001. One-way ANOVA in (**B**,**C**); two-way ANOVA in (**A**,**D**–**G**). BMDM: bone marrow-derived macrophage; SSO: Sulfosuccinimidyl oleate sodium.

To confirm the role of CD36 in EP4-mediated macrophage polarization and foam cell formation, *EP4^MKO^* BMDMs were transfected with CD36-siRNA or NC-siRNA, followed by oxLDL stimulation (Figure S4). In *EP4^MKO^* BMDMs, oxLDL-induced foam cell formation was significantly enhanced; however, such a change was attenuated by CD36 knockdown (Figure 7A). To further assess the interplay between EP4 and CD36 in macrophage polarization, CD36-siRNA-transfected BMDMs were subjected to M1- or M2-polarizing conditions. Upon LPS/IFN-γ stimulation, CD36 knockdown reduced mRNA levels of M1-associated pro-inflammatory factors compared to *EP4^MKO^* BMDMs treated with NC-siRNA (Figure 7B); conversely, IL-4/IL-13 stimulation of CD36 knockdown BMDMs resulted in increased expression of anti-inflammatory genes (Figure 7C).

To assess the functional relevance of CD36 in *EP4^MKO^*-induced atherosclerosis, *ApoE^−/^^−^EP4^Flox^* and *ApoE^−/^^−^EP4^MKO^* mice were fed a Western diet for 12 weeks in vivo, followed by their being intraperitoneally injected with Sulfosuccinimidyl oleate sodium (SSO, CD36 inhibitor) or saline for an additional 4 weeks. Compared to saline-treated controls, SSO administration significantly reduced plaque area in both the whole aorta (Figure 7D) and the aortic root (Figure 7E), decreased intraplaque lipid deposition (Figure 7F), and increased collagen content (Figure 7G). Collectively, these findings demonstrate that EP4 regulates macrophage polarization and foam cell formation through CD36 regulation, thereby contributing to the progression of atherosclerosis.

## 4. Discussion

Atherosclerosis is a chronic vascular inflammatory disease characterized by thickening and hardening of the arterial wall due to lipid deposition and inflammatory response [6,20]. Macrophages play an important role in the progression of AS, particularly through their roles in foam cell formation and phenotypic switching [21,22]. In the present study, we demonstrate that EP4 deficiency enhances macrophage foam cell formation and M1 polarization at least in part by upregulating CD36 expression, thereby exacerbating the progression of atherosclerosis.

EP4 is a PGE2 receptor and is involved in numerous pathophysiological processes [23,24]. While in vitro evidence indicates that EP4 signaling suppresses PGE2-triggered inflammatory responses in macrophages [25], the role of EP4 in vivo remains controversial. While EP4 deficiency has been reported to induce macrophage apoptosis and attenuate early development of atherosclerosis [26], some studies reported that EP4 deficiency has no effect on plaque size or morphology during early stages of AS, but exacerbates local inflammation, and increases the plaque instability during advance stages [27]. In type I diabetic mice, EP4 deficiency in myeloid cells did not significantly affect plaque formation [28]. We herein demonstrate that macrophage-specific EP4 deficiency enhances vascular inflammation, accelerates atherosclerotic progression, and destabilizes plaques during advanced stages of AS. Notably, EP4 has been reported to be upregulated in human atherosclerotic plaques, where it exacerbates inflammation through upregulation of matrix metalloproteinase [25]. In contrast, we found that EP4 expression was significantly downregulated in macrophages of the atherosclerotic plaques. Such discrepancy may be attributed to the differential expression of EP4 in different cell types in atherosclerotic plaques, or EP4 may exert stage-dependent dual roles.

EP4-associated proteins have been found to interact with NF-KB to dampen macrophage activation, acting as a suppressor of inflammation [29]. In the context of type II diabetes, EP4 has also been shown to influence islet inflammation by regulating macrophage polarization [30]. Consistently, our study shows that EP4 deficiency skews macrophage polarization toward the M1 phenotype and enhances pro-inflammatory cytokine production. With respect to lipid metabolism, previous studies have shown that EP4-knockout mice spontaneously develop hypercholesterolemia by regulating the synthesis and elimination of bile acids [16], suggesting the important role of EP4 in lipid homeostasis. In our study, macrophage-specific EP4 deficiency promotes foam cell formation but does not alter plasma cholesterol levels, suggesting that the primary role of macrophage EP4 lies in regulating lipid uptake rather than systemic lipid metabolism.

CD36 is a membrane scavenger receptor that facilitates foam cell formation by mediating cholesterol uptake [31]. In addition, CD36 also promotes macrophage-driven chronic inflammation by mitochondrial metabolic pathways [32]. During atherosclerosis, activated eosinophils can regulate macrophage switching from M2 to M1, which is partly mediated by the CD36 signaling pathway [33]. Consistent with these studies, our findings reveal that CD36 contributes to both lipid accumulation and inflammatory signaling in macrophages, and these processes are regulated by EP4. Interestingly, CD36 has been reported to exert its function through complex formation with other membrane receptors, such as Na/K-ATPase-α1, which exerts a pro-inflammatory effect [34]. In our study, we observed an increased expression of CD36 in EP4-deficient macrophages; however, the precise mechanism by which EP4 regulates CD36 expression remains to be elucidated.

The study has several limitations. First, although we demonstrated that EP4 deficiency promotes macrophage M1 polarization and foam cell formation by upregulating CD36 expression, the precise mechanism by which EP4 regulates CD36 remains unclear and warrants further investigation. Second, since our study was primarily based on a murine model, further studies on clinical samples are needed to determine the significance of EP4-CD36 signaling axis in human atherosclerosis. Third, although the M1/M2 classification has been widely adopted to describe macrophage polarization states, it is increasingly recognized that this binary framework oversimplifies the spectrum of macrophage functional diversity in vivo. Macrophages often display mixed or transitional phenotypes rather than M1/M2. Future studies may further refine macrophage subpopulation characterization through high-dimensional profiling [35,36].

## 5. Conclusions

In summary, our study demonstrates that EP4 attenuates the development of atherosclerosis by regulating CD36 expression, thereby suppressing foam cell formation and M1 macrophage polarization. Targeting the signaling components of the EP4-CD36 signaling axis may provide potential therapeutic targets for AS.

## Data Availability

The original contributions presented in this study are included in the article/Supplementary Material. Further inquiries can be directed to the corresponding author.

## Supplementary Materials

The following supporting information can be downloaded at: https://www.mdpi.com/article/10.3390/cells14131021/s1, Table S1. The primer sequences used in genotyping. Table S2. The primer sequences used in qRT-PCR. Table S3. The sequences for siRNA. Figure S1. EP4 expression is significantly decreased in EP4MKO mice. Figure S2. The EP4 level in HMDMs after siRNA transfection. Figure S3. RNA sequencing analysis of DEGs in BMDMs from EP4Flox and EP4MKO mice following oxLDL stimulation. Figure S4. The CD36 level in BMDMs after siRNA transfection.
