# Supplementary material for: Macrophage EP4 Deficiency Drives Atherosclerosis Progression via CD36-Mediated Lipid Uptake and M1 Polarization
Source: Cells. 2025 Jul 4;14(13):1021. doi: 10.3390/cells14131021 (PMC12249252; doi:10.3390/cells14131021)
Supplement: Supplementary file 1 [file cells-14-01021-s001.zip › cells-3672982-supplementary.pdf]

Supplementary Tables and Figures

Table S1. The primer sequences used in genotyping.

Primer	Forward sequence	Reverse sequence
**EP4 Flox**	GGCGGGATCAGTTAGATGG	GTGAAGCGAGTCCTTAGGC
**Lyz2-cre (mutant)**	CCCAGAAATGCCAGATTACG	
**Lyz2-cre (common)**	CTTGGGCTGCCAGAATTTCTC	
**Lyz2-cre (wild type)**	TTACAGTCGGCCAGGCTGAC	
**ApoE (common)**	GCCTAGCCGAGGGAGACCG	
**ApoE (wild type)**	TGTGACTTGGGAGCTCTGCAGC	
**ApoE (mutant)**	GCCGCCCCGACTGCATCT	

Table S2. The primer sequences used in qRT-PCR.

Primer	Forward sequence	Reverse sequence
**CXCL9 (mouse)**	GACTACATAAGAGACCACTTC	GCCATCCTCCTTTGGAATGATA
**NOS2 (mouse)**	GTTCTCAAGGCACAGGTCTC	GCAGGTCACTTATGTCACTTATC
**β-actin (mouse)**	CGTTGACATCCGTAAAGACC	TAGGAGCCAGAGCAGTAATC
TNF-α **(mouse)**	GGGTGTTCATCCATTCTCTACC	TTGGACCCTGAGCCATAATC
**IL6**	TGTATGAACAACGATGATGCACTT	ACTCTGGCTTTGTCTTTCTTGTTATCT

**(mouse)**		
**IL10 (mouse)**	CAGGGATCTTAGCTAACGGAAA	GCTCAGTGAATAAATAGAATGGGAAC
**Arg1 (mouse)**	TTCTCAAAGGGACAGCCACG	TCAAGCAGACCAGCCTTTCT
**CCL2 (mouse)**	CGCGTCGTGAAACACTTCTA	GATCGGCACAGATCTCCTTAT
**CD206 (mouse)**	GGGTTGCTATCACTCTCTATGC	TTTCTTGTCTGTTGCCGTAGTT

Table S3. The sequences for siRNA.

Gene	Sense (5'-3')	Antisense (5'-3')
**NC-siRNA**	UUCUCCGAACGUGUCACGUTT	ACGUGACACGUUCGGAGAATT
**EP4-siRNA**	UGUCAGUUUUGUCAAAUACCCA	GGGUAUUUGACAAACUGACAUU
**CD36-siRNA**	GUGCCGUAUUGGUAGUUCUTT	AGAACUACCAAUACGGCACTT

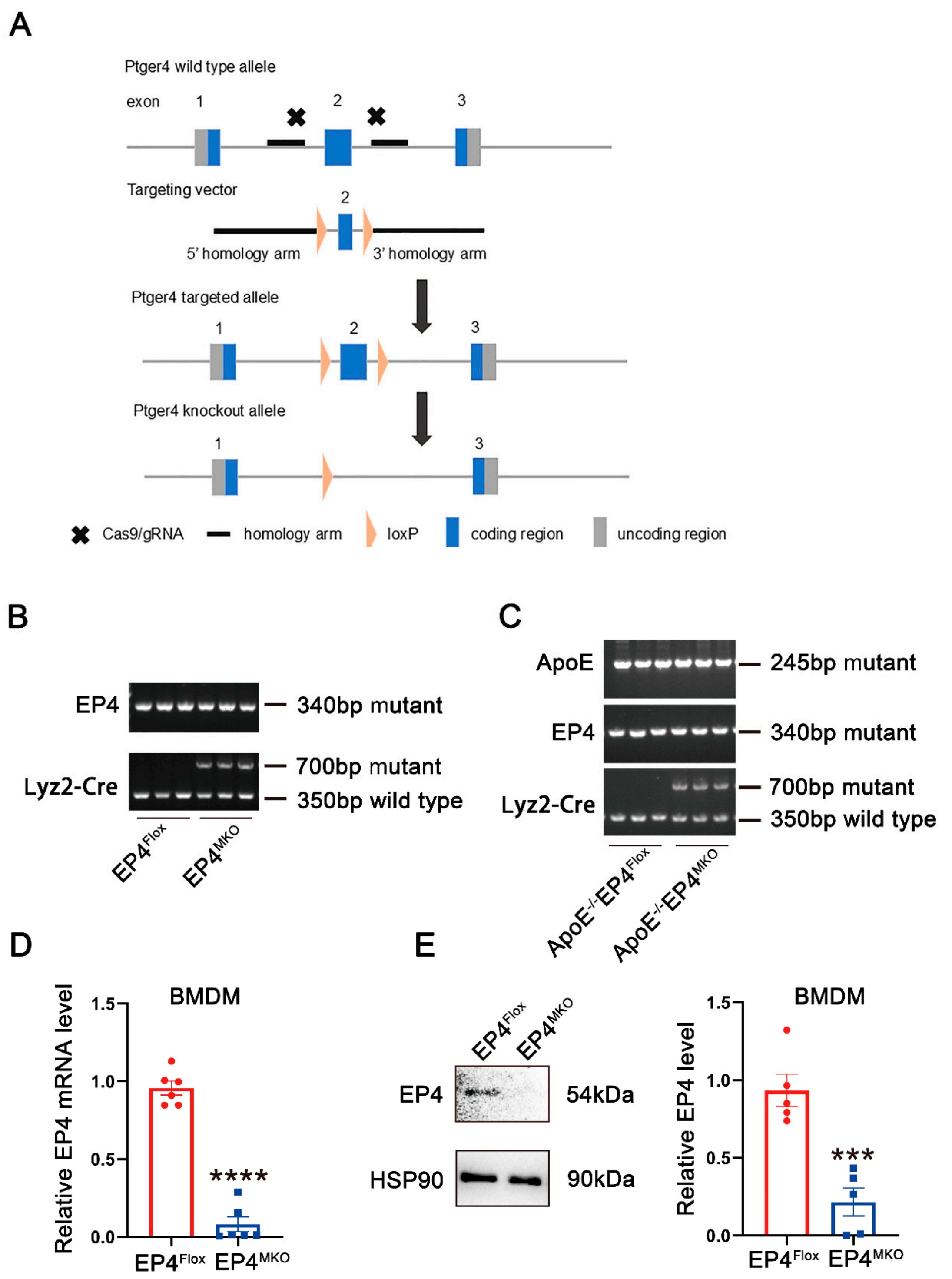

Figure S1. EP4 expression is significantly decreased in EP4^{MKO} mice. (A) Schematic strategy for generating EP4^{Flox} mice. (B) PCR-based genotyping using specific primers. (C) Representative genotyping results for ApoE$^{-/-}$EP4^{Flox} and ApoE$^{-/-}$EP4^{MKO} mice. (D) EP4 mRNA levels in BMDMs isolated from EP4^{Flox} and EP4^{MKO} mice (n=6). (E) EP4 protein levels in BMDMs isolated from EP4^{Flox} and EP4^{MKO} mice (n=5). Data are presented as mean±SEM, $^{***}P<0.05$, $^{****}P<0.0001$, Unpaired t-test.

BMDM: bone marrow- derived macrophage.

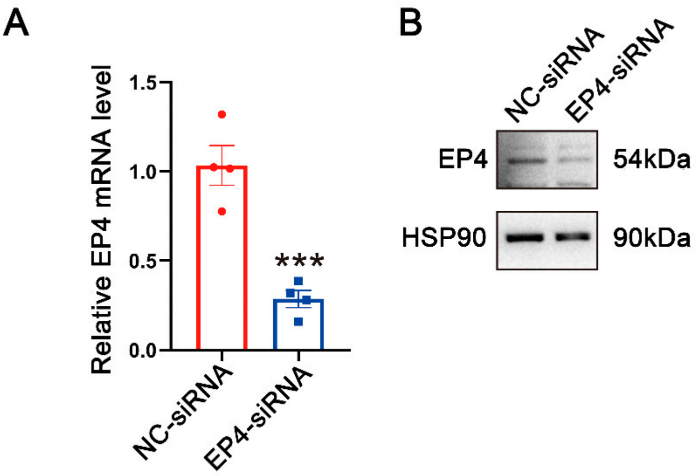

Figure S2. The EP4 level in HMDMs after siRNA transfection. (A) Relative mRNA expression levels of EP4 in HMDMs transfected with NC-siRNA and EP4-siRNA (n=4). (B-C) Western blot analysis and quantification of EP4 protein expression in HMDMs after transfection with NC-siRNA and EP4-siRNA. (n=3). Data presented as mean±SEM, $^{**}P<0.01$, $^{***}P<0.001$, Unpaired *t*-test. HMDM: human monocyte-derived macrophage.

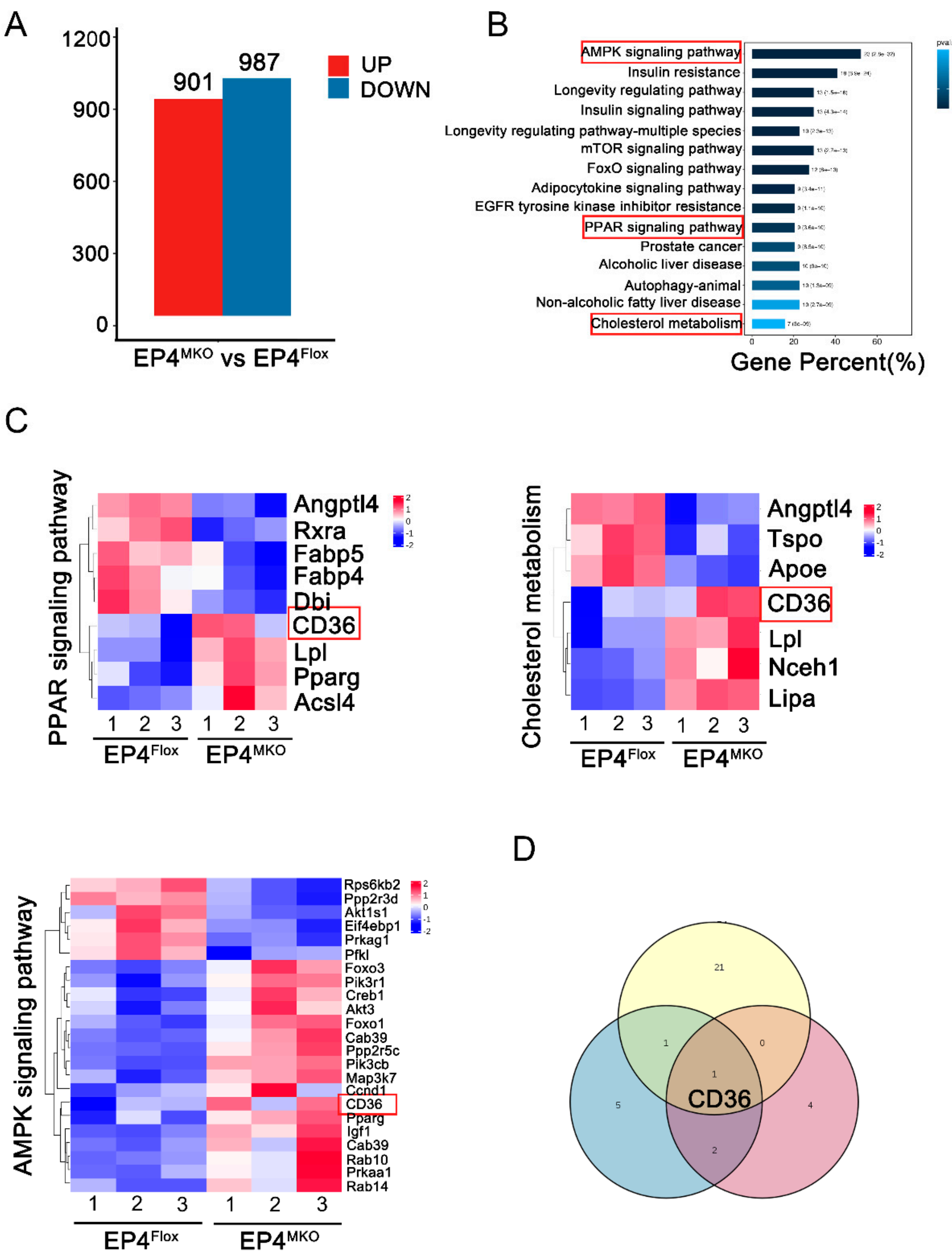

Figure S3. RNA sequencing analysis of DEGs in BMDMs from EP4^{Flox} and EP4^{MKO} mice following oxLDL stimulation. (A) The histogram depicting DEGs in BMDMs from EP4^{Flox} and EP4^{MKO} mice following oxLDL stimulation. (B) The KEGG pathway enrichment analysis of DEGs. (C) The gene sets enriched in each KEGG inflammation and lipid metabolism related pathways. (D) Schematic diagram illustrating the overlap of DEGs among KEGG inflammation and lipid metabolism related signaling pathways.

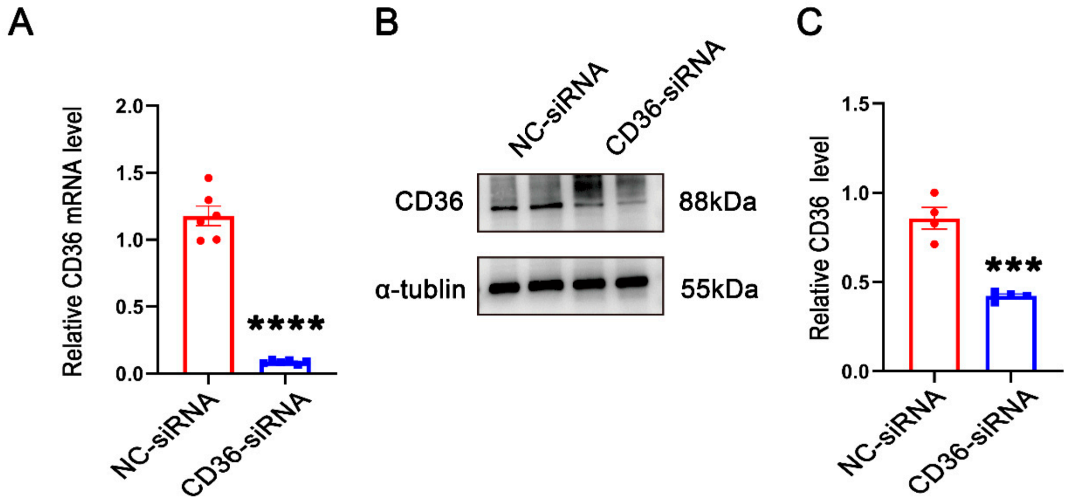

Figure S4. The CD36 level in BMDMs after siRNA transfection. (A) Relative mRNA expression levels of CD36 in BMDMs transfected with NC-siRNA and CD36-siRNA (n=6). (B-C) Western blot analysis and quantification of CD36 protein expression in BMDMs after transfection with NC-siRNA and CD36-siRNA. (n=4). Data presented as mean±SEM, $^{***}P<0.001$, $^{****}P<0.0001$, Unpaired *t*-test. BMDM: bone marrow-derived macrophage.
